# Supplementary material for: Strategies to Connect Low-Income Communities with the Proposed Sewerage Network of the Dhaka Sanitation Improvement Project, Bangladesh: A Qualitative Assessment of the Perspectives of Stakeholders
Source: Int J Environ Res Public Health. 2020 Oct 1;17(19):7201. doi: 10.3390/ijerph17197201 (PMC7579280; doi:10.3390/ijerph17197201)
Supplement: Supplementary file 1 [file ijerph-17-07201-s001.pdf]

**Supplementary Table 1.** Thematic distribution of codes and selection

| Thematic Areas | Categories | Sub-categories | Codes | Responses | | | |
|---|---|---|---|---|---|---|---|
| | | | | FGD | | | KII |
| | | | | Landlord/ homeowners | Tenants | CBO leaders | |
| *Perceptions of current sewerage facilities* | Current sewerage system of Dhaka city | Current sanitation facilities | shared toilet | ✓ | ✓ | ✓ | × |
| | | | narrow lane | ✓ | ✓ | ✓ | ✓ |
| | | | narrow connecting pipes | ✓ | ✓ | × | ✓ |
| | | | no water supply facility inside the toilet | ✓ | ✓ | ✓ | × |
| | | Opportunities | NGO provided toilets | ✓ | ✓ | ✓ | × |
| | | Where faecal matters finally discharged? | storm drainage | ✓ | ✓ | ✓ | ✓ |
| | | | septic tanks | × | ✓ | × | × |
| | Perception regarding sewerage system | Common problems | pipe blockage | ✓ | ✓ | × | ✓ |
| | | | pipe leakage | ✓ | ✓ | × | × |
| | | | Waterlogging | ✓ | ✓ | × | ✓ |
| | | | frequent overflow | × | ✓ | ✓ | × |
| | | | bad odor | ✓ | ✓ | × | × |
| | | | Cholera | ✓ | ✓ | × | × |
| | | | skin disease | × | ✓ | × | × |
| | | Who is responsible? | the government | ✓ | ✓ | ✓ | × |
| | | | DWASA | ✓ | ✓ | ✓ | ✓ |
| | | | NGOs | ✓ | ✓ | ✓ | ✓ |
| | | | Landlords | × | ✓ | ✓ | × |
| *Affordability of having* | Willingness to have sewerage connection | Willingness to connect | a portion of total installation cost | ✓ | × | ✓ | × |
| | | | financial support for toilet improvement | ✓ | × | ✓ | ✓ |

| | | | | | | | |
|---|---|---|---|---|---|---|---|
| *sewerage connection* | | Willingness to pay | household type/size based service charges | × | ✓ | ✓ | ✓ |
| | | | area-based subsidies | × | ✓ | ✓ | × |
| | | | income-based subsidies | ✓ | ✓ | ✓ | × |
| | Co-production | GO/NGO's responsibility | government permission | ✓ | ✓ | ✓ | × |
| | | Landlord/homeowners/community leaders responsibility | negotiate with the authority | × | ✓ | ✓ | × |
| | | | ensure sewerage facilities | × | ✓ | ✓ | × |
| | | | regular monitoring | ✓ | × | ✓ | ✓ |
| | | | pay the tariffs | × | ✓ | × | × |
| | Recommendation | To implementing authority | widen the roads | × | × | ✓ | ✓ |
| | | | wider sewerage pipes | ✓ | × | ✓ | ✓ |
| | | preferred payment methods | monthly bills | ✓ | ✓ | ✓ | ✓ |
| | | | one-time payment | ✓ | × | ✓ | × |
| | | | equated monthly instalments (EMI) | ✓ | × | ✓ | × |
| | | | cash vouchers | ✓ | × | ✓ | × |
| *Challenges and opportunities of connecting LICs to a sewerage network* | Proposed policy | Plan for sewerage connection installation | force to connect (for the houses within 100 meters of proposed trunk main) | × | × | × | ✓ |
| | | Plan for tariffs | subsidized/reduced tariffs | × | ✓ | ✓ | ✓ |
| | Strategies | Possible strategies | Sewerage network on government's cost | ✓ | ✓ | ✓ | ✓ |
| | | | improve existing toilets | ✓ | ✓ | ✓ | ✓ |
| | | | subsidised service charges | ✓ | ✓ | × | ✓ |
| | | | providing loans | ✓ | × | × | ✓ |
| | | Other feasible strategies | on-site sanitation | ✓ | × | × | ✓ |
| | | | communal tank | × | × | × | ✓ |
| | | | separating industrial waste lines | × | × | × | ✓ |
| *Barriers of being* | Problem issues | Major risks | growing populations | × | × | × | ✓ |
| | | | high-rise buildings | × | × | × | ✓ |

| | | | | | | | |
|---|---|---|---|---|---|---|---|
| *connected with the sewerage network* | | | narrow roads | ✓ | ✓ | ✓ | ✓ |
| | | | overlapping of various utility connections | × | × | × | ✓ |
| | | Major challenges | 20% coverage | × | × | × | ✓ |
| | | | old sewerage network | ✓ | × | × | ✓ |
| *Perceived benefits of sewer connection* | Perceived benefits | environmental benefits | Safe disposal | ✓ | ✓ | ✓ | ✓ |
| | | | no bad odour | ✓ | ✓ | ✓ | × |
| | | | no clogged drain | ✓ | × | ✓ | ✓ |
| | | | safe drinking water | ✓ | ✓ | ✓ | × |
| | | | no contact with wastewater | ✓ | ✓ | × | × |
| | | | no overflow | ✓ | ✓ | × | × |
| | | health benefits | no contact with pathogens | ✓ | ✓ | × | × |
| | | | no contamination | ✓ | ✓ | ✓ | ✓ |
| | | | no mosquitoes and flies | ✓ | ✓ | × | × |
| | | | no breathing problems | ✓ | ✓ | ✓ | × |
| | | | no skin diseases | ✓ | ✓ | × | × |

**Ticks and crosses beside the codes indicating that which codes were frequently mentioned by which groups of the particip.
